# A Systematic Review of the Sport Psychology Mixed Martial Arts Literature: Replication and Extension

**DOI:** 10.3390/ejihpe12020007

**Published:** 2022-01-19

**Authors:** Sydney Cooper, Marc Lochbaum

**Affiliations:** 1 Honors College, Texas Tech University, Lubbock, TX 79409, USA; sydneyco@ttu.edu; 2 Department of Kinesiology and Sport Management, Texas Tech University, Lubbock, TX 79409, USA; 3 Education Academy, Vytautas Magnus University, 44248 Kaunas, Lithuania

**Keywords:** UFC, performance psychology, mental toughness, social facilitation, mental skills, mood

## Abstract

MMA is a global sport with a growing body of psychological literature. Our main objective was to replicate and extend a past review concerning the sport psychology literature with MMA participants. We conducted our electronic search in EBSCO with the following databases: SPORTDiscus, PsycINFO, ERIC, and Psychology and Behavioral Sciences Collection. Our eligibility criteria were research articles (a) found in academic journals, (b) with MMA participants, and (c) at least one topic found in sport psychology literature. After conducting a PRISMA-guided search, 16 studies met our inclusion criteria. The studies spanned from 2011 to 2021, with 795 MMA participants from the USA (n = 7), Brazil (n = 4), and one study each from Czechia, Poland, Spain, Sweden, and the United Kingdom. From studies reporting mean ages, MMA participants were in their mid-20s (M = 26.55 ± 2.38 years of age). The results section includes risk of bias ratings across five areas (i.e., subject selection, sample’s MMA background, participant anonymity, data collection procedures, and questionnaire or qualitative theme reporting). More risk of bias concerns resulted with the quantitative than qualitative articles. To best represent the studies, we presented separate results tables with many specifics for both the quantitative (i.e., topic, main analysis, time frame, summary of results, and meaningfulness) and qualitative studies (topic, main analysis, time frame, and main themes). The included studies covered a variety of historic and meta-analyzed topics such as confidence, mood, motivations, and social facilitation. Based on our review, we discussed the literature strengths and limitations, and suggested future research directions. Last, we provided practical points for both MMA participants and their trainers.

## 1. Introduction

If, indeed, Koch’s [1] publication titled *Calisthenics from the Viewpoint of Dietetics and Psychology* is the first sport psychology publication [2], then sport psychology is nearing 200 years as an academic discipline. Since Koch [1], sport psychology research and professional practice has flourished across sports and countries. Mixed martial arts (MMA) compared to the history of sport psychology is a new sport with the first Ultimate Fighting Championship, later titled UFC 1: The Beginning, on 12 November 1993, in Denver, Colorado. As with all sports, mental characteristics [3,4], mood [5,6,7], and use of sport psychology techniques [8,9,10] matter to achieve in sport in addition to physical requirements. Indeed, of Issurin’s [11] seven categories required for elite athletic performance, some categories include sport psychology topics such as mental toughness, intrinsic motivation, self-regulation, and discipline. There is no reason to believe these sport psychology topics and many others fail to apply to MMA fighters.

Though a recent review exists on sport psychology with MMA athletes [12], we believed the review to be deficient in a few areas. First, though thought of as recent with a 2020 publication date, the submission date was 24 November 2017. The MMA sport psychology literature seems to be growing. Hence, several years have passed since the authors conceived their review and searched for articles. Second, within the period of their search, we believed more articles existed. Given the number of articles in their review (N = 8), a few missed articles might lead to an improved representation of the MMA sport psychology research literature. Third, the authors stated following PRISMA guidelines. However, they did not provide an assessment of individual study risks of bias. Last, a better understanding of the quantitative data is possible. Though our review is not a meta-analysis, meaningfulness statistics exist in published articles or can be calculated, and thus, improve our understanding of the MMA and sport psychology literature beyond statistical significance.

### 1.1. History of MMA and Sport Psychology

When trying to find the origin of MMA as a stand-alone in the sport psychology literature, Harpold’s [13] published thesis seems the first. Harpold examined the mental skills of six amateur MMA athletes in training and competition. Harpold stated his purpose was to investigate the required thoughts and perceptions for successful MMA fighting. Harpold research was qualitative within a humanistic framework. Once interviewed privately over the phone or in person, the research team (Harpold formed a group to help with his qualitative data analyses) went through several phases to find relevant data and to distinguish the overlying themes. The resultant themes were confidence, visualization and mental rehearsal, arousal regulation, mental toughness, and motivation. Each of the themes has more in-depth sub themes, for example, self-talk, scouting, and breath control. Through the interviewing process, the researchers found a plethora of positive outcomes in the themes and sub themes from the consistencies of the MMA athletes’ experiences. Thus, undoubtedly and as expected, MMA athletes use sport psychology techniques and discuss often research topics such as confidence [14] and mental toughness [15].

Since Harpold’s research, enough published research followed for Andrade and colleagues [12] to conduct a systematic review. Their systematic review included eight studies fitting their eligibility criteria. The included studies addressed fear, aggression, emotional control, confidence, mental toughness, motivation, arousal, coping, rational emotive behavioral therapy for MMA athletes, fighting experience or MMA competition. Professional athletes comprised seventy-five percent of Andrade and colleagues’ included studies. When examining the study methodologies, the majority (n = 5) were qualitative studies. Andrade et al. concluded the following four main points.

1. Fighters who relied on making money from the sport had higher amounts of stress.
2. All MMA athletes, of the varying levels, the greatest fears were losing or becoming injured.
3. MMA fighters try to subdue their fears by treating every fight as “another day in the office” and by using positive self-talk.
4. MMA is continuously growing, unfortunately the lack of sports psychology research done on the competitors and sport is self-defeating because the fighters will not recognize the importance of the psychology.

### 1.2. Objectives

As mentioned before, though informative, we believed given the popularity of MMA via the rise of the UFC and the abundant sport psychology literature for centuries, more articles must exist within and past Andrade and colleagues’ [12] review period, which ended in 2017. In addition, we noted a few other omissions such as reporting meaningfulness in the studies with qualitative data. Hence, our main objective was to pull together the psychological literature found with MMA participants to replicate and extend the work of Andrade and colleagues’ [12]. In doing so, we aimed to report on the quality, contents, limitations, and provide concrete future directions based on the reviewed literature.

## 2. Materials and Methods

Each applicable PRISMA [16] statement guided our review. To fulfill the primary funded research commitment, we formulated the search inclusion criteria and overall research plan prior to writing this manuscript.

### 2.1. Eligibility Criteria

We included articles meeting the following criteria: (a) articles found in a peer-reviewed academic journal with no language restriction; (b) participants involved in MMA; and (c) topic found in the sport psychology literature. We based the participants’ involvement in MMA on specifics found in the study participant descriptions. We did not consider participants outside the realm of MMA such as boxing, judo, and karate.

### 2.2. Information Sources and Search Process

**Figure 1:**
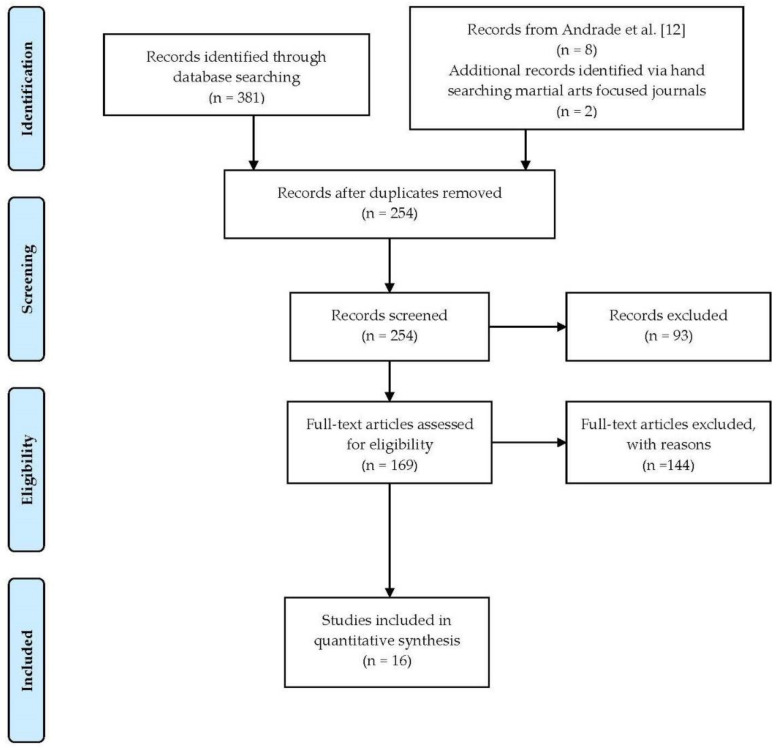
PRISMA flowchart.

After verifying Andrade and colleagues’ [12] included articles, we began our search. Our electronic search occurred in EBSCO following the PRISMA [17] search process (see Figure 1) with the following individual databases: ERIC, Psychology and Behavioral Sciences Collection, PsycINFO, and SPORTDiscus. Both authors participated in the EBSCO database search, which concluded on 22 March 2021. The second author extensively examined the search and expanded the search to 10 June 2021. Mixed martial and psychology were our search terms. We used the EBSCO advanced search option that provided three separate boxes for search terms such as box 1 (mixed martial), box 2 (psychology) and box 3 (N/A). At each stage, we restricted EBSCO to a five-year period (e.g., 2010–2015). Once a five-year period was exhausted, each author restarted with the next year (e.g., 2015–2020). Here are the details of one five-year period search strategy that resulted in four articles with none selected:

1. Delimited search to 2004–2009,
2. Box 1 typed in mixed martial, and
3. Box 2 typed in psychology.

We also searched reference lists of found EBSCO articles. In addition, two journals (*Martial Arts Studies* found at https://mas.cardiffuniversitypress.org/about/ and *Revista de Artes Marciales Asiaticas* found at http://revpubli.unileon.es/ojs/index.php/artesmarciales/about) caught our attention as needing to be hand searched (last accessed date 10 June 2021). The second author completed these hand searches and corresponded with the first author as to the contents.

### 2.3. Data Collection Process and Items

To understand the studies and perform our replication and extension of the MMA sport psychology literature, we extracted the following information: (a) the citation, (b) country of study, (c) sample information (size, sample percent males, mean age or age range, sample experience level, source of the participants), (d) author stated objective and topic, (e) methodological information (main analyses, type of data collected, time frame of study), and (f) results (author stated and any effect size data presented). We collected additional information as found in the *Risk of Bias in Individual Studies* section. Both authors conducted the data extraction by him or herself. Then, we compared our extractions, discussed, and formed consensus.

### 2.4. Risk of Bias in Individual Studies

We coded for individual study risk of bias as appropriate given the mix of qualitative and quantitative studies. We aimed to determine if the quality of included studies (i.e., low risk of bias) varied and potentially influenced our discussion. Given the study of MMA in sport psychology is mostly likely not the result of randomized clinical trials [12], we examined a number of risk bias examples found in the literature [18] spanning both internal and external validity issues as opposed to a clinical trials type of coding system. We coded all studies on the following risks of bias: (a) subject selection; (b) sample’s MMA background; (c) participant anonymity; (d) data collection procedures; and (e) questionnaire or qualitative theme reporting.

### 2.5. Planned Methods of Analysis and Summary Measures

Though our review is systematic and not both systematic and meta-analytic, we sought to provide statistics such as means, correlations, and higher-order themes. If some of these pulled statistics and themes were similar, we planned to combine them to provide the best overall review of the included studies or at least provide a discussion of meaningfulness for each study. We used the Comprehensive Meta-Analysis (CMA) version-3 software (version 3.3.070, Biostat, Inc., Englewood, NJ, USA, 20 November 2014) to calculate mean difference effect size values (i.e., Hedges’ *g*) when an effect size was not found in the included quantitative articles. For correlation interpretation, we utilized Cohen’s [19] guidelines: above 0.50 as large, between 0.30 and 0.49 as moderate, and between 0.10 and 0.29 as small. For mean difference interpretation, we followed Cohen’s [20] interpretation: above 1.30 as very large, between 0.80 and 1.29 as large, between 0.50 and 0.79 as medium, and between 0.20 and 0.49 as small. For partial eta squared (η2) interpretation, we founded the following guidelines [21]: above 0.14 as large, between 0.06 and 0.13 as medium, and between 0.01 and 0.05 as small.

## 3. All Study Results

### 3.1. Study Characteristics

Table 1 includes the 16 [13,22,23,24,25,26,27,28,29,30,31,32,33,34,35,36] studies meeting our inclusion criteria. The studies spanned from 2011 to 2021, with 795 MMA participants (n mean = 49.68 ± 63.65, range 3 to 215) with data coming from North America (USA, n = 7), South America (Brazil, n = 4), and one study each from five European countries (Czechia, Poland, Spain, Sweden, and the United Kingdom). The majority (n = 12) of the studies [13,22,24,25,26,28,29,30,31,34,35,36] included only male MMA athletes. From studies reporting mean ages, MMA participants were in their mid-20s on average (M = 26.55 ± 2.38 years of age). One study [29] included two large samples (n = 618; n = 278) of non-MMA participants (approximately 65% females) as raters of the MMA participants. Of the eight articles not found in the Andrade et al. (2020) review, five [13,26,28,29,33] articles were found within their search timeframe and three [23,27,35] beyond their search timeframe.

### 3.2. Study Quality Ratings

Table 2 includes our individual study bias concerns across the following five categories: subject selection, sample background, participant anonymity, data collection procedures, and questionnaire or qualitative theme reporting. We noted some concern in just over half of the studies regarding subject selection. Given MMA research is not the level of clinical based research with random selection and the like, the subject selection procedures overall seemed adequate, especially with the number of professional including UFC athletes as participants. Hence, for our second bias category concerned with the sample’s MMA background, the vast majority (n = 13) we rated from no (n = 4) [23,29,34,35] to low (n = 3) [22,24,30] to some (n = 6) [25,27,28,31,32,33] concern. Though it is not a requirement to state participant anonymity assured, it seems relevant. Most likely, researchers assured anonymity within their human subject participant forms. However, only three studies [23,29,35] specially wrote anonymity assured in their method section. Concerning data collection, most studies (n = 12) collected their data directly from their participants. Only, Chen and Cheesman [25] collected data via an online survey. Concerning our last bias category, questionnaire or qualitative theme reporting, we found no need for concern with the qualitative studies [30,31,32,33,34,35,36]. We found concern with the quantitative studies, as more than half failed [22,26,27,28] to report study level reliability. Overall, we found the quantitative studies of greater risk for bias than the qualitative studies.

### 3.3. Quantitative Studies

Table 3 (topic, main analyses, and study design) and Table 4 (result summary and meaningfulness) contain specifics for all studies [22,23,24,25,26,27,28,29,30] with quantitative data. Mood was the only repeated topic [24,28], but not with the same objective. Both studies appeared to utilize the Portuguese version [37] of the Brunel Mood Scale [38]. The topics were stress and coping [22], social facilitation [23], weight loss and mood [24], mood difference based on competition schedule [28], mental toughness [25], talent development requirements [27], and motivations for starting MMA [26]. Perceptions of aggression (and relation to competitive success) based on facial features was the other topic, with non-competitive MMA fighters as the raters [29]. Last, in the mixed-methods study, Cunningham and Turner [30] reported on self-referenced beliefs and acceptance data of MMA athletes. All studies except one [29] presented mean level data, and two [23,25] presented both mean level and correlational. Many studies [22,25,26,27,28,29] were cross-sectional, though a few [24,30] spanned at least a month. The Blomqvist Mickelsson and Shaw’s [23] study utilized archival data over a three-year period.

Concerning result interpretation (see Table 4), we sought to report effect size values, author reported or calculated or interpreted (e.g., reported correlations) by us. Specifically, MMA fighters whose income depends on fighting reported greater stress than those whose income does not solely depend on fighting [22]. In addition, in Belem, regardless of income needs, MMA fighters who had a diverse set of coping strategies had better physical and psychological recovery during competitions. Though insufficient data existed to calculate precise effect sizes for these result as the authors reported Spearman rank correlations or limited mean difference statistics, examination of the presented data suggest small effects in all Belem’s summarized results.

In the first of the two mood studies [24,28], competitive MMA fighters who used rapid weight loss strategies produced greater results (i.e., more weight loss) than those without a strategy with this weight loss accompanied by increased confusion and total mood disturbance [24]. We interpreted the mood changes as small with author reported effect size values for the significant findings. Continuing with the theme of mood, Silva [28] found that tension and anger increases when the MMA athletes have a scheduled fight. We calculated mean difference effect sizes for tension and anger, as well as fatigue and confusion. Averaged together, the effect size was medium, with the upper end of the 95% confidence intervals being large. Hence, we believe Silva and colleague’s [28] data might be more important than reported.

Three studies [25,26,27] examined often-researched psychological constructs, namely mental toughness, talent and excellence characteristics, and motivation. Regarding mental toughness, Chen and Cheesman [25] reported data on superior athletic performance and enhanced mental toughness. Chen and Cheeseman viewed their results supporting the research literature suggesting superior athletic performance and performers possess greater mental toughness than their less superior competitors do. Based on the effect size data presented by the authors [25], the differences were medium between MMA professionals and non-professionals on three mental toughness components (i.e., determination, positive cognition, and confidence). In the same line of reasoning as Chen and Cheesman [25], Ruiz-Barquin and colleagues [27] examined psychological characteristics required to develop excellence. They concluded superior psychological abilities for developing excellence as characteristics of MMA competitors compared to athletes of other sports, those being long-term support, use of imagery, and quality practice. We calculated effect size values between the MMA sample and the other archival sport data sets. The effect sizes were medium, with the 95% confidence interval ranging from medium to large. Kuśnierz et al. [26] examined a variety of motivation factors for beginning MMA training. They reported the most prominent being enjoyment from training entertainment. Other motivating factors included the benefit of the sport for improvement of health and enjoyment of learning new fighting techniques. We found insufficient data reported for effect size calculations. In addition to specifics to MMA enjoyment, the authors [26] reported MMA athletes (and the boxing participants) self-reported greater internal compared to external motivations for their sports.

Blomqvist Mickelsson and Shaw [23] and Třebický et al. [29] uniquely studied MMA and sport psychology topics by way of archival MMA data. Blomqvist Mickelsson and Shaw [23] interpreted their findings that the presence of an audience impaired MMA fighters’ performances. Though we found insufficient data to calculate effect size values, our visual inspection of their frequency graph suggested to us a large effect. It appears audiences do indeed impair MMA fighter performances when compared to the COVID-19 pandemic audience, less fights. Třebický et al. [29] likewise used existing MMA competitor data (faces and fight records). After having facial features rated by a large sample of on-MMA participants, the authors reported perceptions of aggression might relate to success. The reported correlation was small; thus, the success of MMA fighters used in the study related to success, albeit small in magnitude.

Last for the quantitative data reported, Cunningham and Turner [30] reported participants 2 and 3 (participant 1 did not complete the study) in their mixed-methods study had post and 6-month post treatment reductions in total rational beliefs and self-depreciating thoughts. The differences ranged from small to medium in meaningfulness.

### 3.4. Qualitative Studies

Table 5 contains specifics (topic, main analysis, and study duration) for studies [13,30,31,32,33,34,35,36] with qualitative data. Based on the author stated objective and our reading of the individual articles, the topics were traditional sport psychology topics [13,32,33,36], aggression/violence [34,35], REBT therapy [30], and the fighting experience [31]. The researcher methodologies varied a bit. They described the following as the approaches: phenomenological [13,31,35], grounded theory [32,34], reversal theory [34], ethnography [36], and note reflections [30]. Half of the studies interviewed the participants once [13,31,34,35] while the other saw and interacted with the participants from at least 6 months [30], to a year [32,33] and finally for two years [36].

As with our quantitative studies, we sought to interpret the author stated results with more details, specifically with our finding and reporting their higher-order themes. Table 6 contains the specifics. Harpold [13] sought to ask his amateur MMA athletes about their mental skills use. Five themes emerged concerned with confidence, arousal regulation, imagery, mental toughness, and motivation. Massey and colleagues [32,33] with extensive notes, observations, and interviews over one year with nine MMA athletes including some who competed in the UFC examined psychological training and competition factors and self-regulation strategies to enhance performance. Massey et al. [32] reported five higher-order themes concerned self-regulation as a process of motivation, both internal and external, and ongoing evaluation. The researchers [32] also reported MMA athletes deliberately induce pain and mental distress as part of their psychological training. Massey and colleagues [33] with the same participants reported behavioral processes, embodied emotions, and psychological strategies are invaluable for enhanced performance. Our summary of Massey et al. [33] included higher-order themes such as self-liberation, counter conditioning, stimulus control, contingency management, and helping relationships. Last, of the psychological skills focused qualitative studies, Vaccaro et al. [36] over two years with 215 competitive MMA athletes, examined fear management. Their [36] summary was fighters feared injury and losing, with the fear of both needing management. More experienced fighters were capable of concealing and controlling their fear, as newer competitors tended to struggle because of the lack of strategies/experience [36]. The four higher-order themes centered on personal fear to using the fear as a performance strategy (i.e., fostering fear).

For the two studies examining aggression/violence, Rosario et al. [34] interviewed professional MMA fighters. Given the violent nature of combat sports, the MMA fighters viewed aggression as an important mood state for success when used as a strategy. We pulled five themes from Rosario and colleagues’ work concerned with aggression defined, importance, influential factors, use in training versus competition, and some reversal theory concepts. Serrano Rodrigues and colleagues [35] examined the psychology of violence in fighting. As with Rosario et al. [34], we reported five overall themes from Serrano Rodrigues et al. Those themes included knowing when to use violence/aggression in matches as opposed to using violence/aggression without focus and disproportionately. Unquestionably, and again logical to combat sports, violence/aggression is required for success. The interviewed MMA fighters reported the need to master the use of violence/aggression for success.

First of the two unconnected studies, Jensen et al. [31], interviewed MMA competitors, amateur and professionals, concerning the most important factors within their fighting experience. Jensen et al. reported the chaotic nature was the most important experience. The higher-order themes were the cage reality, fighting skill, purpose, and community. The community theme we took at the sport psychology nature of Jensen and colleagues’ work. The qualitative aspect of Cunningham and Turner’s [30] mixed-methods study reported participants’ ability to change self-depreciation and acceptance thoughts. The previously discussed quantitative data seemed of more value to understanding the value of REBT. Certainly, the study notes are also of value. For instance, participants reflected on other MMA athletes, seeing how anxiety was a limiting fault and the need to decrease such negative thoughts. In addition, the participants changed their perception of enhanced athletic performances by gaining self-confidence in themselves by respecting their strengths and their opponent’s weaknesses.

## 4. Discussion

In our introduction, we suggested that whatever we found, Issurin’s [11] psychology of elite performers topics would apply. Furthermore, given Issurin’s psychological categories and subtopics span historical and mostly meta-analyzed constructs, i.e., [3,4,5,6,7,8,9,10]; we expected some of those topics in the MMA-specific sport psychology literature. Those constructs indicated by Issurin as characteristic of elite performers such as confidence [13], motivation [26], imagery 13], goal setting [22], mental toughness [13,25], and mood states [24,28], appeared and seemed of importance to MMA athletes. In addition, the search pulled up articles focused on talent development [27], self-regulation defined in many ways [33], and competitive strategies concerned with aggression [34]. Stemming from the global COVID-19 pandemic, social facilitation, a historic topic [39] and meta-analyzed many decades ago [40], gained research attention [23]. Given our initial stated aim and subsequent review, we believe we replicated and more vital extended the understanding of MMA and sport psychology literature.

Even with our expectations and our knowledge from an MMA sport psychology focused review [12], we did not know the possible breadth of sport psychology topics once we completed our search and review. Though sport science reviews on physiological research with a specific population [41] and a specific sport task and with sport psychology topics [42] exist in the literature, not having the knowledge of the possible breath is one of our limitations. The outcome of few overlapping topics and none with similar data measurement purposes made meta-summary difficult and no determination of whether study quality (individual bias risks) influenced our summary. Along with this main limitation, as with the Andrade and colleagues [12], the search process even with the benefit of electronic databases never seems perfect. Given the global nature of MMA and the potential for MMA population-specific articles to be found in unique journals as we found, missed articles in our search is another possible limitation.

Though limitations exited in our review, several strengths exist in the 16 included studies. First, some participants were elite (i.e., UFC) MMA fighters as well as lower level professionals and participants with international experiences. The archival studies, of course, allowed for UFC fighters as participants [23] or the focus [29] of the investigation. Another strength was the quality of the qualitative studies [13,30,31,32,33,34,35,36]. The qualitative studies included many long-term projects, spanning from at least 6 months [30] to two full years of detailed fieldwork [36]. Massey and colleagues [32] first qualitative study appeared in *Psychology of Sport and Exercise*, a premier sport psychology journal and the official journal of the European Federation of Sport Psychology. Last as an important strength, though seemingly with more risk of bias concerns than the qualitative studies, some of the quantitative studies presented effect size data to assist in discussing meaningfulness of the results as opposed to only statistical significance.

Even with the strengths, limitations are part of the research process at both the individual study and overall review levels. At the individual study level, a few limitations are worth discussion. The first limitation is the reporting of the questionnaire data. Authors [22,26,27,28] as well as the journal editors failed to provide study specific reliability values. All of these studies appear to be using translated questionnaire versions. Given this, we suggest journals require at the minimum reliability values and perhaps should require confirmatory factory analyses of the questionnaire. Of course, during the review process, it is possible that the authors provided this information to the journal editor and reviewers. However, given the international interest up to the time of our search (i.e., Brazil, Czechia, Poland, Spain, and Sweden) in sport psychology with MMA athletes, journals must demand more questionnaire rigor.

Reporting exact participant expertise level is another potential limitation and thus area for improvement. Swann and his colleagues [43] provided taxonomy for classifying athletic samples. They based their taxonomy, including both within and between sport comparisons, on their extensive thematic review of 91 studies between 2010 and 2013. We suggest the use of this taxonomy in all research and of course with sport psychology research with MMA athletes. Authors did seem to note when UFC athletes were participants. The UFC seems unquestionably to be the highest recognized MMA expertise level. Last worth discussion, though we followed the PRISMA guidelines for systematic reviews, missed literature may occur. We found the sport psychology MMA literature at times confusing as to ethnographic works in the sociology literature. For instance, Spence [44] interviewed 45 MMA athletes regarding injury and pain relating to the athletes’ masculine identity. Certainly, athletic identity [45] is a sport psychology topic. Works such as Spence’s, as defined by the author, is sociological in nature.

## 5. Conclusions

MMA and sport psychology is an evolving literature. To date, researchers addressed historic and often studied sport psychology themes. In addition, given the violent nature of MMA, researchers examine aggression and fear. MMA participants can gain insight as to the importance of mental skills by reading our review. For instance, our review suggests MMA participants should dedicate time to learning self-regulation skills, explore their fears, and understand the potential financial stress of MMA as a career. Likewise, trainers of MMA fighters should understand from our review that they should emphasize mental skills within the talent development process. Though not mature and often studied, the MMA and sport psychology studies are indeed interesting and of great methodological variety, given the literature contains research endeavors spanning cross-sectional to two years and archival research spanning three years. However, no one researcher or research group appears dedicated to a continued line of MMA and sport psychology studies. Hence, as MMA continues to evolve as a sport and a sport with research attention, being aware of the published literature included in our review is of value for researchers to determine next steps for a focused and sustainable research agenda.

## Figures and Tables

**Table 1:** Sample country, sample size (N), percent male participants, participant age mean or age range, participant experience, and source of participants for all reviewed studies.

REF #	Country	N	Male%	Age	Experience	Participant Source
[22]	Brazil	50	100	25.00	National and international	Identified training sites
[23] **	Sweden	86	84.88	NR	Professional (UFC)	Sherdog.com (last accessed 7 July 2021)
[24]	Brazil	12	100	25.60	National	Mato Grosso State
[25]	UK	136	100	27.10	Amateur, semi-professional, and professional	UK based Cage Warriors forum
[26] *	Poland	23	100	24.30	Not stated	Opole sport clubs
[27] **	Spain	42	78.57	26.21	Amateur, semi-professional, and professional	Not stated
[28] *	Brazil	40	100	26.00	Amateur, regional, national, and international	Academies in Florianópolis region
[29] *	Czechia	146	100	29.77	Professional (UFC)	www.ufc.com (last accessed 7 July 2021)
		618	34.95	26.46	Perceived aggressiveness raters	Czech Republic
		278	35.25	27.52	Perceived fighting ability raters	Czech Republic
[30]	USA	3	100	23.67	Semi-professional	Coach forwarded to research team
[13] *	USA	6	100	20 to 30	Amateur	3 USA cities
[31]	USA	7	100	31.40	Amateur and professional	Recommended by MMA promoters and trainers at city-level MMA associations
[32]	USA	9	88.90	NR	Amateur and professional including UFC	An MMA academy
[33] *	USA	9	88.90	NR	Amateur and professional including UFC	An MMA academy
[34]	USA	6	100	23 to 30	Professional	Authors’ MMA network
[35] **	Brazil	5	100	23 to 34	Professional	Combat sport gyms, cites in São Paulo state
[36]	USA	215	100	26.50	Competitive, exact level not stated	First author contact with MMA gym

Notes: * = article found within Andrade et al. review timeframe, ** = article found outside Andrade et al. (2020) search time frame, and NR = not reported.

**Table 2:** Bias concern areas (bias resulting from…) for all included studies.

REF #	Subject Selection	Sample Background	Participant Anonymity	Data Collected Procedures	Questionnaire or Qualitative Theme Reporting
[22]	Some	Low	Some	No	Some
[23]	Some	No	N/A	N/A	N/A
[24]	Some	Low	Some	No	No
[25]	Some	Some	Some	Moderate	No
[26]	Some	Moderate	Some	No	Some
[27]	Some	Some	Some	No	Some
[28]	Some	Some	Some	No	Some
[29]	Low	No	Some	No	N/A
[30]	Some	Low	Some	Low	No, Moderate
[13]	Some	High	Low	Low	No
[31]	Low	Some	Low	No	No
[32]	Low	Some	Some	No	No
[33]	Low	Some	Some	No	No
[34]	Low	No	Low	No	No
[35]	Low	No	Some	No	No
[36]	Low	Moderate	Some	No	No

Notes: Subject selection: Low = purposeful recruitment or large sample size; Some = random selection did not occur. Sample background: No = professionals (e.g., UFC); Low = national; international; Some = amateurs; semi-professionals; Moderate = level not stated; high = amateurs only. Participant anonymity: N/A = not applicable; Low = anonymity assured; some = anonymity not stated. Data collection procedures: N/A = not applicable; No = in person data collection; Low = mix in person and online; Moderate = data collected online. Questionnaire or Qualitative Theme Reporting: N/A = not applicable; No = questionnaire reliabilities reported or multiple researchers reviewed transcripts; Some = questionnaire reliabilities not reported; Moderate = therapy not verifiable.

**Table 3:** Topic, main analysis, and study time frame for studies with quantitative results.

REF #	Topic(s)	Main Analyses	Study Design
[22]	Coping strategies, stress	Correlation, mean difference	Cross-sectional
[23]	Social facilitation	Mean difference	Archival
[24]	Mood	Mean difference	Repeated measures
[25]	Mental toughness	Correlation, mean difference	Cross-sectional
[26]	Motivation	Mean difference	Cross-sectional
[27]	Talent, excellence characteristics	Mean difference	Cross-sectional
[28]	Mood	Mean difference	Cross-sectional
[29]	Perception	Correlation, multivariate regression	Cross-sectional
[30]	Self-depreciation, acceptance beliefs	Mean differences, diary keeping, rational emotive therapy notes, reflections	Intervention

**Table 4:** Result summary and meaningfulness for studies with quantitative results.

REF #	Summary	Meaningfulness
[22]	Financial dependence is stressful. More training associated with increased use of goal setting and adversity confrontations. Coping strategies improve in competition recovery.	Insufficient data reported and the use of Spearman rank correlations for exact effect size calculations. With information provided, all results appear small.
[23]	Presence of audience can impair performance.	Insufficient data reported for exact effect size calculations. Visual inspection of frequency graph suggest presence of audience was large.
[24]	Rapid weight loss disrupts mood.	Author reported effect size values corresponding with *p* values < 0.05 all small (range 0.16–0.22).
[25]	Mental toughness characteristic of better performing athletes.	Author reported partial eta squared values medium for mental toughness subscales determination (0.07), positive cognition (0.12), and confidence (0.12) for professionals vs. non-professionals.
[26]	Enjoyment main reason for starting training. Internal greater than external motivation.	Insufficient data reported for any effect size calculations or estimates to verify author stated results.
[27]	Long-term support, use of imagery, and quality practice are perceived as important for talent development.	Calculated Hedges’ g values from Table 1 data support overall medium in meaningfulness differences, 0.62 [95% CI 0.41–0.84] between the MMA participants and all other participants on factors I, II, and IV.
[28]	Daily anger and stress increase with upcoming fight.	Calculated Hedges’ g values from author provided values (in text) support medium on average, 0.51 [95% CI 0.18–0.84] differences, overall with the individual values being tension (0.54), anger (0.54), fatigue (0.47), and confusion (0.49) between those with scheduled fights and those without.
[29]	Facial features associated with aggression might increase competition success.	Author reported correlation between perceived aggressiveness and actual fighting success was small (r = 0.20).
[30]	REBT applicable to MMA athletes.	Author interpreted differences (via Parker and Vannest, 2009 AB single-case effect size suggestions) as small to medium dependent variable changes.

**Table 5:** Topic, main analyses, and study time frame) for studies with qualitative results.

REF #	Topic(s)	Main Analyses	Study Duration
[30]	Self-Depreciation and Acceptance Beliefs	MD, diary keeping, rational emotive therapy notes, reflections	6 months
[13]	Mental Skills	Phenomenological	Cross-sectional
[31]	Perceived Fight Experience	Phenomenological	Cross-sectional
[32]	Psychological Factors	Grounded theory including participant observations, field notes, and interviews	1 year
[33]	Self-Regulation	Constructivist philosophy including participant observations, field notes, and interviews	1 year
[34]	Aggression	Grounded theory, reversal theory	Cross-sectional
[35]	Transition of Violence	Phenomenological	Cross-sectional
[36]	Fear Management	Ethnography including note taking, informal and formal interviews, and training with the participants	2 years

**Table 6:** Main themes for studies with qualitative results.

REF #	Main Theme(s)
[30]	REBT applicable to MMA athletes
[13]	(1) Confidence, (2) arousal regulation, (3) imagery and mental rehearsal, (4) discipline/mental toughness, and (5) motivation
[31]	(1) Cage reality, (2) purpose, (3) fighting skill, and (4) community
[32]	(1) Self-regulation: motivation and ongoing evaluation; (2) external factors of self-regulation and performance; (3) creation and maintenance of an ascetic routine; (4) internal factors of self-regulation and performance; and (5) deliberately induced physical pain and psychological distress
[33]	(1) Self-liberation, (2) counter conditioning, (3) stimulus control, (4) contingency management, and (5) helping relationships
[34]	(1) Definitions of aggression; (2) importance of aggression; (3) factors influencing aggression; (4) aggression in training versus competition; and (5) reversal theory concepts
[35]	(1) Maintaining focus vs. losing your head; (2) maintenance of the technique vs. loss of the technique; (3) feeling good vs. embarrassment; (4) desire to test yourself vs. aggression; and (5) desirable retaliation vs. disproportionate retaliation
[36]	(1) The fears of fighting, (2) fighting fear, (3) framing, and (4) fostering fear

## Data Availability

All data coding sheets are available from the corresponding author.
